# Effects of Dietary Phosphate on Adynamic Bone Disease in Rats with Chronic Kidney Disease – Role of Sclerostin?

**DOI:** 10.1371/journal.pone.0079721

**Published:** 2013-11-13

**Authors:** Juliana C. Ferreira, Guaraciaba O. Ferrari, Katia R. Neves, Raquel T. Cavallari, Wagner V. Dominguez, Luciene M. dos Reis, Fabiana G. Graciolli, Elizabeth C. Oliveira, Shiguang Liu, Yves Sabbagh, Vanda Jorgetti, Susan Schiavi, Rosa M. A. Moysés

**Affiliations:** 1 Department of Internal Medicine, Nephrology Division, Universidade de São Paulo, São Paulo, Brazil; 2 The Sanofi-Genzyme R&D Center, Genzyme Corporation, Framingham, Massachusetts, United States of America; University of Milan, Italy

## Abstract

High phosphate intake is known to aggravate renal osteodystrophy along various pathogenetic pathways. Recent studies have raised the possibility that dysregulation of the osteocyte Wnt/β-catenin signaling pathway is also involved in chronic kidney disease (CKD)-related bone disease. We investigated the role of dietary phosphate and its possible interaction with this pathway in an experimental model of adynamic bone disease (ABD) in association with CKD and hypoparathyroidism. Partial nephrectomy (Nx) and total parathyroidectomy (PTx) were performed in male Wistar rats. Control rats with normal kidney and parathyroid function underwent sham operations. Rats were divided into three groups and underwent pair-feeding for 8 weeks with diets containing either 0.6% or 1.2% phosphate: sham 0.6%, Nx+PTx 0.6%, and Nx+PTx 1.2%. In the two Nx+PTx groups, serum creatinine increased and blood ionized calcium decreased compared with sham control group. They also presented hyperphosphatemia and reduced serum parathyroid hormone (PTH) and fibroblast growth factor 23 (FGF23) levels. Fractional urinary excretion of phosphate increased in Nx+PTx 1.2% rats despite lower PTH and FGF23 levels than in sham group. These biochemical changes were accompanied by a decrease in bone formation rates. The Nx+PTx 1.2% group had lower bone volume (BV/TV), higher osteoblast and osteocyte apoptosis, and higher SOST and Dickkopf-1 gene expression than the Nx+PTx 0.6% group. Nx+PTx 0.6% rat had very low serum sclerostin levels, and Nx+PTx 1.2% had intermediate sclerostin levels compared with sham group. Finally, there was a negative correlation between BV/TV and serum sclerostin. These results suggest that high dietary phosphate intake decreases bone volume in an experimental model of CKD-ABD, possibly via changes in SOST expression through a PTH-independent mechanism. These findings could have relevance for the clinical setting of CKD-ABD in patients who low turnover bone disease might be attenuated by optimal control of phosphate intake and/or absorption.

## Introduction

The chronic kidney disease associated mineral and bone disorder (CKD-MBD) is characterized by complex endocrine and metabolic disturbances, with a wide variability in terms of bone turnover, ranging from extremely low to extremely high bone formation rates [1]. Adynamic Bone Disease (ABD) as one extreme of the different forms of renal osteodystrophy has become an increasingly common manifestation of bone abnormalities in CKD patients. ABD may be associated with serious clinical consequences such as fractures [2,3] and vascular calcification [4,5], which in turn may contribute to the high mortality rates of patients with CKD. 

The hallmark of ABD is a decrease in bone turnover together with normal or low osteoid surface [1,6–10]. The pathogenesis of ABD linked to CKD is multifactorial, including old age, diabetes mellitus, uremic toxins, and excessive suppression of secondary hyperparathyroidism by high calcium input via the dialysate or calcium-containing phosphate binders, pharmacological doses of active vitamin D sterols or parathyroidectomy [11,12]. At the cellular level, resistance to parathyroid hormone (PTH) secondary to PTH receptor downregulation and decreased osteoblast number and activity are prevalent features. The latter results from reduced osteoblast proliferation and enhanced apoptosis, which are important factors in the determination of bone formation rates [11]. However, a clear understanding of the molecular mechanisms that lead to ABD, as well as the potential role of other bone cell types, in particular the osteocyte, is still lacking. 

Osteocytes synthesize bone remodeling factors including the receptor activator of nuclear factor kappa-B ligand (RANKL), osteoprotegerin (OPG) and sclerostin and thus participate in the control of osteoclast and osteoblast activity [13–16]. Recent studies have suggested that the Wnt/β-catenin signaling pathway, which is expressed not only in the osteoblast, but also in the osteocyte, plays a role in the regulation of normal bone turnover [17] and in renal osteodystrophy [18].

Wnt/β-catenin pathway inhibitors such as sclerostin, encoded by SOST gene and produced by mature osteocytes, and Dickkopf-1 (Dkk-1), encoded by Dkk-1 gene and expressed by a variety of cells [19], antagonize Wnt/β-catenin canonical signaling and thereby lead to decreased bone formation [20]. A recent study has shown that the serum levels of both inhibitors are elevated in hemodialysis patients, with an inverse correlation of serum sclerostin with serum PTH and bone formation rate [21]. 

The effects of PTH on bone may be mediated, at least partly, by changes in sclerostin expression. Exogenous administration of PTH has been shown to result in downregulation of osteocytic sclerostin expression, both *in vivo* and *in vitro* [22,23]. In addition to PTH, other factors are possible regulators of SOST gene expression, like decreased mechanical loading [24]. 

Whether suppression of PTH secretion contributes to CKD-associated ABD via changes in Wnt/β-catenin pathway activity has not been investigated. Although ABD in CKD patients occurs most frequently in the context of low or normal serum PTH levels it can also be observed in presence of high PTH levels [25]. Therefore, factors other than PTH clearly play a role as well, including calcium and phosphate overload. In order to gain a more detailed insight into the pathogenesis of this disease, we induced ABD in an experimental rat model of hypoparathyroidism combined with chronic kidney failure. Our main purpose was to examine the influence of the uremic state itself and the importance of phosphate overload.

## Materials and Methods

This study was carried out in strict accordance with the recommendations in the Guidelines of the standing Committee on Animal Research of University of São Paulo. The protocol was also approved by the Committee on the Ethics of Animal Experiments of University of São Paulo (Permit Number: 0962/08). All surgery was performed under pentobarbital anesthesia, and all efforts were made to minimize suffering. 

### Experimental protocol

Male Wistar rats, initial body weight 300-350 g, were obtained from our local breeding colony for use in this study. They were housed in individual cages in a light-controlled environment (12 h on/12 h off), at constant temperature (25°C) and humidity (25%) and fed a standard diet (Lab Diet 5002, Purina Mills, USA), containing phosphate (0.6%), Ca (0.8 %), protein (20%) and vitamin D3 2.2 IU/g, for one week. Thereafter, they were anesthetized with pentobarbital (50 mg/kg I.P.) and divided into three groups. Two groups underwent total parathyroidectomy (PTx), involving microsurgical technique using electrocautery, and 5/6 nephrectomy (5/6 Nx) as described previously [26]. A third group underwent sham operation (sham Nx+sham PTx). One day after surgery, animals were divided into three groups and allocated to different diets: Nx+PTx 0.6%, which received a 0.6% phosphate diet (Lab Diet 5002, Purina Mills, USA); Nx+PTx 1.2% which received a 1.2% phosphate diet (Modified Lab Diet 5002 w/1.2% P, USA) and sham group (sham Nx+PTx), which received a 0.6% phosphate diet (Lab Diet 5002, Purina Mills, USA). Thus all diets had same composition of Ca, protein and vitamin D_3_, except for phosphate content. A pair-feeding protocol was used, where the amount of feed provided to the pair of animals was determined by the animal of the pair that had eaten less food. Weight measurements and tail cuff plethysmography recordings were performed weekly. Water access was *ad libitum*. The study duration was 8 weeks. A fluorochrome bone marker (Terramycin®) at a dose of 25 mg/kg was injected I.P. on days 11 and 12, as well as on days 4 and 5 before sacrifice. For the last two days of the study, the rats were held in metabolic cages and 24 h urine samples were collected. Eight weeks after surgery, rats were anesthetized and sacrificed through aortic puncture exsanguination. Serum samples were frozen at - 20°C for later biochemical evaluation. The heart was excised and left ventricle dissected for weight. Femurs were removed for bone histomorphometry and tibiae were removed for evaluation of osteoblast and osteocyte apoptosis and gene expression. 

### Biochemical analysis

Serum and urinary creatinine and phosphate (colorimetric assay, Labtest, Lagoa Santa/MG, Brazil), blood ionized calcium (iCa) (AVL-9140 Autoanalyzer AVL Scientific Corporation, Roswell, GA, USA), urinary calcium (colorimetric assay, Cobas, Roche, Indianapolis, IN, USA), serum immunoreactive PTH (iPTH, ELISA, Immutopics, San Clemente, CA, USA), fibroblast growth factor 23 (FGF23) (ELISA assay, Kainos Laboratories, Tokyo, Japan), serum sclerostin (ELISA, EIAab Science Co. Ltd., China) and serum calcitriol (1,25 OH_2_ vitamin D_3_) (1,25-Dihydroxy Vitamin D RIA, Immunodiagnostic Systems, Boldon, United Kingdom) were measured. Albuminuria was determined by radial immunodiffusion method [27].

### Bone histomorphometry

At sacrifice, the left femur of each rat was removed, dissected free of soft tissue, immersed in 70% ethanol, and processed as described previously [28]. Static, structural and dynamic parameters of bone formation and resorption were measured in distal metaphyses (magnification, 250x; 30 fields), 195 µm from the epiphyseal growth plate, using an Osteomeasure image analyzer (Osteometrics, Atlanta, GA, USA). Structural parameters included trabecular thickness, trabecular separation (expressed in µm) and trabecular number (expressed as number/mm). Static parameters included ratios of trabecular volume/bone volume, osteoid volume/bone volume, osteoid surface/bone volume, osteoblast surface/bone volume, fibrosis volume, eroded surface/bone surface, osteoclast surface/bone surface, all expressed as percentages, and osteoid thickness, expressed in µm. Mineral apposition rate was determined from the distance between the two terramycin labels, divided by the time interval between the two terramycin administrations and expressed in µm/day. Mineralization lag time was expressed in days. The percentage of double terramycin-labeled (mineralizing) surface per bone surface and bone formation rate completed the dynamic evaluation. Histomorphometric indices were reported using nomenclature recommended by the American Society of Bone and Mineral Research [29]. All animal data were obtained through with the examiners blinded to the study protocol. 

### Osteoblast and osteocyte apoptosis

Apoptosis was determined in the left tibia by TUNEL technique (TdT-mediated X-dUTP Nick end labeling, using the instructions provided by Apoptag plus Peroxidase in Situ Apoptosis Detection Kit. To evaluate the percentage of apoptotic osteoblasts and osteocytes in the cortical and trabecular areas as well as in bone marrow, we used the counting points method. Each cell type was analyzed in 60 fields, with a magnification of 1.000x, to obtain final values expressed as percent apoptotic cells.

### Gene expression analysis

The right tibia of each rat was used to measure gene expression of SOST, Dkk-1, β-catenin, glycogen synthase kinase 3 beta (Gsk3b), lymphoid enhancer-binding factor 1 (Lef1), low density lipoprotein receptor-related protein 5 (Lrp5), low density lipoprotein receptor-related protein 6 (Lrp6), **s**ecreted related-frizzled protein 1 (Srfp1), **s**ecreted related-frizzled protein 4 (Srfp4), transforming growth factor beta 1 (TGF-β1), Wnt 7b, Wnt 10b, OPG, RANKL, Receptor activator of nuclear factor kappa B (RANK), and RANKL/OPG, as analyzed by TaqMan Low Density Array (TLDA) technique. These genes are related to the activity of osteoblasts and osteoclasts and the Wnt/β-catenin pathway, Srfp1 and Srfp4 being inhibitors of this pathway.

For these analyses, bones were harvested and snap frozen in Trizol (Sigma, St. Louis, MO, USA). Bone shafts were collected, epiphyses removed, bone marrow separated via centrifugation, and the shafts placed in Trizol (Sigma, St. Louis, MO, USA). RNA was extracted using the chloroform and isopropanol precipitation method. The extracted RNA was treated with DNase, purified on a Qiagen (Valencia, CA, USA) column and eluted in RNAse free water. A reverse transcriptase reaction was performed subsequently. The generated cDNA was used in single Taqman assays or Taqman low density arrays (Applied Biosystems, Carlsbad, CA, USA) containing genes of interest and assayed according to the manufacturer’s protocol. The difference in expression was calculated using 18S as the control gene.

### Statistical analysis

Results are presented as mean ± standard deviation (SD) or as median (interquartile ranges). One-Way ANOVA and Kruskal Wallis test were used for parametric and nonparametric data respectively. A linear regression test (Spearman) was used to assess the correlation between two variables. GraphPad Prism software, version 4.0 (GraphPad, San Diego, CA, USA) was used. P values <0.05 were considered statistically significant.

## Results

### General data

Initial body weight was comparable among the 3 rat groups. Nx+PTx groups had lower food intake, lower final body weight, higher tail cuff pressure (TCP) and higher heart weight compared to sham group. We did not observe differences in food intake, final body weight, TCP or heart weight between Nx+PTx 0.6% and Nx+PTx 1.2% groups (Table 1). 

**Table 1:** General data.

	**Sham**	**Nx+PTx 0.6%**	**Nx+PTx 1.2%**
	**(n=10)**	**(n=9)**	**(n=9)**
Initial BW (g)	253 ± 33	247 ± 13	273 ± 29
Final BW (g)	378 ± 10	320 ± 35 ^a^	312 ± 32 ^a^
Food intake (g/day)	19 (18-20)	17 (14-18) ^a^	18 (17-18) ^a^
TCP (mmHg)	114 ± 6	143 ± 8 ^a^	137 ± 10 ^a^
HW/BW	0.17 (0.17-0.19)	0.21 (0.18-0.26) ^a^	0.25 (0.21-0.26) ^a^

TCP: Tail cuff pressure. HW/BW: heart weight/100 g body weight; Nx+PTx: 5/6 nephrectomy and total parathyroidectomy. a: p <0.05 vs. sham.

### Laboratory findings

As shown in Table 2, Nx+PTx rats had lower creatinine clearance (Ccreat), with correspondingly higher serum creatinine, and phosphate levels and higher albuminuria, as well as markedly lower blood iCa and serum FGF23 levels than sham group. Fractional excretion of phosphate phosphate FE was higher in Nx+PTx 1.2% than in the other groups. Serum calcitriol and calciuria did not differ between the three groups. Nx+PTx groups had 4-10 times lower median PTH levels than sham group but the differences did not reach statistical significance. There were no differences between Nx+PTx groups in regards to serum creatinine, phosphate, FGF23 and PTH, blood iCa, Ccreat, and albuminuria. Finally, serum sclerostin was lower in Nx+PTx 0.6% group than Nx+PTx 0.12% and sham groups, despite similar serum phosphate levels in Nx+PTx groups. 

**Table 2:** Biochemical data.

	**Sham**	**Nx+PTx 0.6%**	**Nx+PTx 1.2%**
	**(n=10)**	**(n=9)**	**(n=9)**
Serum creatinine (mg/dl)	0.6 ± 0.1	1.4 ± 0.9 ^a^	1.3 ± 0.2 ^a^
Ccreat (ml/min)	2.5 ± 0.7	1.0 ± 0.6 ^a^	0.8 ± 0.2 ^a^
Albuminuria (mg/24h)	0.4 (0.3-3.2)	47 (17-147) ^a^	61 (11-104) ^a^
Serum phosphate (mg/dl)	5.5 ± 0.6	12.2 ± 1.9 ^a^	11.7 ± 1.9 ^a^
Phosphate FE (%)	7.6 (3.6-10.2)	1.0 (0.3-9.8) ^b^	42.4 (32.1-54.3) ^a^
Blood iCa (mmol/L)	1.16 (1.07-1.22)	0.46 (0.40-0.52) ^a^	0.50 (0.41-0.60) ^a^
Calciuria (mg/24h)	1.4 (0.6-5.6)	5.1 (2.7-5.8)	1.5 (1.1-2.2)
Serum FGF23 (pg/ml)	286 ± 92.2	192 ± 41.8 ^a^	137.5 ± 111.7 ^a^
Serum 1,25(OH)_2_vitamin D_3_ (pg/ml)	16.1 ± 9.0	54.6 ± 0.0	23.3 ± 16.9
Serum PTH (pg/ml)	124 (88-199)	26 (19-117)	16 (5-224)
Serum sclerostin (ng/ml)	1.71 (0.71-3.35)	0.15 (0.07-0.43) ^a^,^b^	1.10 (0.51-2.66)

Ccreat: creatinine clearance; P: phosphate; phosphate FE: urinary fractional excretion of phosphate; iCa: blood ionized calcium; FGF23: fibroblast growth factor 23; PTH: parathyroid hormone; Nx+PTx: 5/6 nephrectomy and total parathyroidectomy; a: p <0.05 vs. sham; b: p<0.05 vs. Nx+PTx 1.2%.

### Bone histomorphometry

As shown in Table 3, both Nx+PTx rat groups showed lower osteoid volume, osteoid surface, osteoblastic and osteoclastic surfaces, eroded surface, mineralization surface, mineral apposition rate, and bone formation rate relative to control sham animals, and there was no fibrosis. These findings confirmed the achievement of a low bone remodeling status.

**Table 3:** Rat bone static and dynamic histomorphometry.

**Bone Parameters**	**Sham**	**Nx+PTx 0.6%**	**Nx+PTx 1.2%**
	**(n=10)**	**(n=9)**	**(n=9)**
BV/TV (%)	24.2 ± 5.01	33.60 ± 4.4 ^a^,^b^	26.81 ± 5.53
OV/BV (%)	0.54 ± 0.47	0.09 ± 0.04 ^a^	0.14 ± 0.09 ^a^
O.Th (μm)	1.30 ± 0.45	1.13 ± 0.18	1.44 ± 0.36
OS/BS (%)	8.15 (3.71-25.57)	2.32 (1.93-2.76) ^a^	2.45 (0.85-6.55) ^a^
ES/BS (%)	14.70 ± 5.35	6.45 ± 2.60 ^a^	5.10 ± 2.38 ^a^
Ob.S/BS (%)	7.07 (4.0-16.75)	1.9 (1.35-2.38) ^a^	2.21 (0.75-5.03) ^a^
Oc.S/BS (%)	2.92 (1.97-5.11)	1.05 (0.53-1.55) ^a^	0.91 (0.40-1.07) ^a^
Fb.V (%)	0	0	0
Tb.Sp (μm)	190.38 ± 46.02	120.14 ± 18.89 ^a^,^b^	161.60 ± 38.95
Tb.N (/mm)	4.13 ± 0.73	5.59 ± 0.52 ^a^,^b^	4.70 ± 0.85
Tb.Th (μm)	58.71 ± 18.66	60.18 ± 5.49	57.19 ± 9.12
MAR (μm/day)	1.1 ± 0.30	0.20 ± 0.12 ^a^	0.68 ± 0.35 ^a^
MS/BS (%)	5.33 ± 3.11	0.75 ± 0.25 ^a^	2.08 ± 0.75 ^a^
BFR/BS (μm^3^/μm^2^/day)	0.054 ± 0.041	0.0016 ± 0.0013 ^a^	0.014 ± 0.0091 ^a^
MLT (day)	3.0 (1.5-3.9)	18.85 (18.7-18.9) ^a^,^b^	1.9 (1.4-11.8)
Aj.AR (µm/day)	0.53 ± 0.28	0.08 ± 0.05 ^a^,^b^	0.68 ± 0.48

BV/TV: trabecular bone/total volume; OV/BV: Osteoid volume/ bone volume; O.Th: Osteoid thickness; OS/BS: osteoid surface/bone surface; ES/BS: eroded surface/bone surface; Ob.S/BS: osteoblast surface/bone surface; Oc.S/BS: osteoclast surface/bone surface; Fb.V: fibrosis volume; Tb.Sp: trabecular separation; Tb.N: trabecular number; Tb.Th: trabecular thickness; MAR: mineral apposition rate; MS/BS: mineralization surface/bone surface; BFR/BS: bone formation rate/bone surface; MLT: mineralization lag time; Aj.AR: Adjusted apposition rate; Nx+PTx: 5/6 nefrectomy and total parathyroidectomy. Nx+PTx: 5/6 nephrectomy and total parathyroidectomy; a: p <0.05 vs. sham; b: p<0.05 vs. Nx+PTx 1.2%.

Nx+PTx 0.6% group had higher bone volume with a corresponding lower trabecular separation and higher trabecular number than Nx+PTx 1.2% group and sham group, respectively. In addition, Nx+PTx 0.6% animals also showed higher mineralization lag time and lower adjusted apposition rate than the two other animal groups (Table 3).

**Figure 1:**
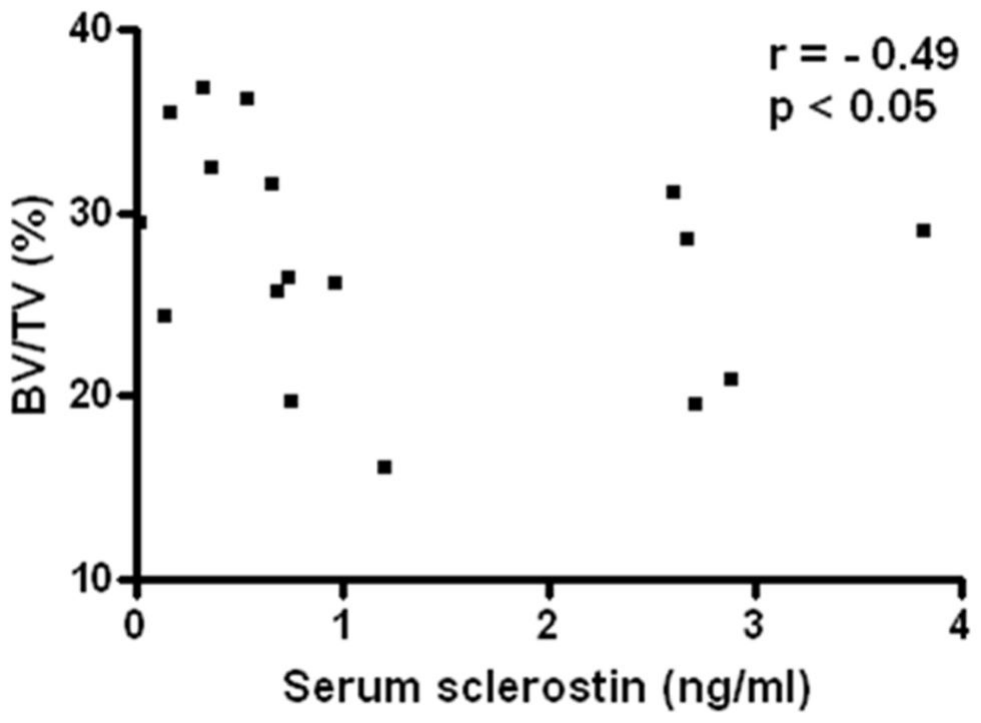
Correlation of serum sclerostin and bone volume (BV/TV) among sham, Nx+PTx 0.6% and Nx+PTx 1.2% groups. Nx+PTx: 5/6 nephrectomy and total parathyroidectomy.

We did not observe any differences in eroded surface or osteoclastic surface between Nx+PTx groups (Table 3). Interestingly, a negative correlation between bone volume and serum sclerostin was found (Figure 1). 

### Osteoblast and osteocyte apoptotic rate

Nx+PTx 0.6% rats had a lower osteoblastic and osteocytic apoptotic rate compared to sham group. The percentage of apoptotic osteocytes and osteoblasts was higher in Nx+PTx 1.2% than in Nx+PTx 0.6% group (Table 4). 

**Table 4:** Osteoblastic and osteocytic apoptotic rates (%).

	**Sham**	**Nx+PTx 0.6%**	**Nx+PTx 1.2%**
	**(n=10)**	**(n=9)**	**(n=9)**
Osteoblast apoptosis, %	1.2 (0.9-1.8)	0.4 (0.3-0.8) ^a^,^b^	1.3 (0.9-1.5)
Osteocyte apoptosis, %	0.5 (0.3-1.0)	0.2 (0.15-0.25) ^a^,^b^	0.4 (0.3-0.5)

Nx+PTx: 5/6 nephrectomy and total parathyroidectomy a: p < 0.05 vs. sham; b: p < 0.05 vs. Nx+PTx 1.2%.

### Gene expression analysis

As shown in Table 5, Nx+PTx 1.2% group had higher SOST and Dkk-1 mRNA levels relative to Nx+PTx 0.6% and sham animals. There were no significant differences in gene expression of β-catenin, Lef1, Lrp6, Srfp1, TGF-β1, OPG, RANKL and RANKL/OPG across any groups. Gsk3b and RANK expression was reduced in Nx+PTx 0.6% relative to Nx+PTx 1.2% and sham groups. Nx+PTx 0.6% also showed lower Lrp5, Srfp4 and Wnt 10b expression than sham group. Nx+PTx 1.2% showed higher Wnt 7b expression than sham animals. 

**Table 5:** Gene expression analysis.

	**Sham**	**Nx+PTx 0.6%**	**Nx+PTx 1.2%**
	**(n=4)**	**(n=6)**	**(n=3)**
SOST	1.2 ± 0.85^b^	0.53 ± 0.24^b^	4.1 ± 0.60
Dkk-1	1.18 ± 0.73^b^	0.86 ± 0.35^b^	2.9 ± 0.72
β-catenin	1.06 ± 0.38	1.26 ± 0.52	0.94 ± 0.06
Gsk3b	1.01 ± 0.17	0.64 ± 0.22 ^a^,^b^	1.17 ± 0.02
Lef1	1.07 ± 0.45	0.72 ± 0.52	0.69 ± 0.04
Lrp5	1.06 ± 0.40	0.45 ± 0.12 ^a^	0.75 ± 0.02
Lrp6	1.02 ± 0.22	0.77 ± 0.24	1.15 ± 0.14
Srfp1	1.02 ± 0.23	0.69 ± 0.36	0.90 ± 0.07
Srfp4	1.11 ± 0.62	0.24 ± 0.08 ^a^	0.34 ± 0.04
Wnt 7b	1.18 ± 0.74	0.11 ± 0.06	0.08 ± 0.04 ^a^
Wnt 10b	1.22 ± 0.73	0.26 ± 0.14 ^a^	0.64 ± 0.16
TGF-β1	1.03 ± 0.29	1.25 ± 0.84	1.13 ± 0.04
OPG	1.09 ± 0.45	0.52 ± 0.30	0.65 ± 0.09
RANKL	1.09 ± 0.49	0.61 ± 0.12	0.71 ± 0.12
RANK	1.02 ± 0.24	0.62 ± 0.23 ^a^,^b^	1.1 ± 0.04

Data are expressed as X control gene and as mean ± SD. Dkk-1: dickkopf-1; Gsk3b: glycogen synthase kinase 3 beta; Lef1: lymphoid enhancer-binding factor 1; Lrp5: low density lipoprotein receptor-related protein 5; Lrp6: low density lipoprotein receptor-related protein 6; Srfp1: **s**ecreted related-frizzled protein 1; Srfp4: **s**ecreted related-frizzled protein 4; Wnt 7b: wingless-type MMTV integration site family, member 7B; Wnt 10b: wingless-type MMTV integration site family, member 10B; TGF-β1: transforming growth factor beta 1; OPG: Osteoprotegerin; RANKL: Receptor activator of nuclear factor kappa B ligand; RANK: Receptor activator of nuclear factor kappa B; RANKL/OPG rate: Receptor activator of nuclear factor kappa B ligand/ Osteoprotegerin rate. Nx+PTx: 5/6 nephrectomy and total parathyroidectomy. a: p <0.05 vs. sham; b: p<0.05 vs. Nx+PTx 1.2%.

## Discussion

In this study, we evaluated the effects of dietary phosphate on ABD in rats with CKD and hypoparathyroidism. Compared to sham group, Nx+PTx rats had higher serum creatinine, lower Ccreat, and hyperalbuminuria consistent with the induction of CKD. Importantly, both Nx+PTx groups had hyperphosphatemia, with no differences between them. However, Nx+PTx 1.2% rats had an increase in phosphate FE subsequent to the higher phosphate load. PTx resulted in blood iCa levels which were reduced by more than half, and was effective in preventing the usual CKD-associated hyperparathyroidism. Hypocalcemia was taken as evidence of successful extirpation of the parathyroid glands, as already described by other authors [30,31]. However, the inhomogeneous reduction of serum PTH levels in the PTx+Nx animals is probably due to low sensitivity of the PTH assay in the hypoparathyroid range, or it is due to hypocalcemia, that may have stimulated some small remnant gland in some animals, since PTH levels were measured after 8 weeks of PTx. Nevertheless, histomorphometry demonstrated low bone remodeling, dismissing any possibility of bone effects of hyperparathyroidism. Decreased levels of serum FGF23 resulted from PTx and this observation is in agreement with previously reports [32,33]. Our results show that dietary phosphate overload, can stimulate phosphate FE even when both PTH and FGF23 levels are decreased. It suggests that a different renal tubule regulation may exist, separate from a change in luminal phosphate delivery, in a situation of ABD, hypoparathyroidism and phosphate overload. Certainly, future studies are needed to evaluate this mechanism, since little is known about the crosstalk between intestine and other organs, in the setting of ABD.

The two Nx+PTx rat groups presented lower bone turnover. However, Nx+PTx 1.2% group had reduced bone volume compared to Nx+PTx 0.6% group. Induction of bone loss by high phosphate intake has been described in normal individuals [34,35] and animals [36]. In CKD patients, studies did not have shown association between P and osteoporosis. Tani et al. [37] demonstrated in mature rats that prolonged exposure to dietary phosphate excess induces bone loss associated with secondary hyperparathyroidism. However, we have previously reported lower bone volume in Nx+PTx rats fed with high P diet (1.2%) that developed hyperphosphatemia in presence of normal PTH. These findings suggest that PTH elevation is not absolutely necessary for phosphate-associated reductions in bone volume [26]. Furthermore, the phosphate effect on bone was independent of PTH infusion rate [38]. 

In addition, the increase in bone volume observed in Nx+PTx 0.6% group, as compared to Nx+PTx 1.2% group, may be secondary to surgical hypoparathyroidism, which leads to an imbalance between resorption and formation in favor of the latter, resulting in increased bone mass at both cortical and trabecular sites, in humans [39]. However, such findings may only partially apply to the present animal study because of the concomitant presence of CKD. 

An alternative mechanism may be the involvement of Wnt/β-catenin pathway in the pathogenesis of CKD-MBD. Because of low PTH levels in the present study, we expected to observe high serum sclerostin levels in both Nx+PTx groups. However, only when the phosphate intake was very high, namely in the Nx+PTx 1.2% group, did SOST mRNA expression increase to higher values than normal and did serum sclerostin levels return to the normal range. The differences between the two CKD rat groups were observed despite similar serum phosphate, PTH and creatinine levels, suggesting that dietary phosphate directly or indirectly regulates β-catenin activity, at least partially through modulation of SOST gene activity. The observed inverse correlation between serum sclerostin and bone volume is consistent with the well-known role of β-catenin in bone mass regulation. Supporting our results, a recent study with predialysis CKD patients showed that phosphate FE and serum Sclerostin levels were elevated at baseline. After therapy with the phosphate binder sevelamer, a decrease in serum Sclerostin was seen despite a significant decrease in serum PTH, suggesting the role of dietary phosphate in the modulation of sclerostin production [40].

The increase in osteoblast and osteocyte apoptosis in Nx+PTx rats in response to high phosphate intake confirms a previous *in vitro* study by Meleti et al [41] in osteoblast-like cells. However, apoptosis could also be mediated by SOST since canonical Wnt signaling appears to protect against programmed cell death through β-catenin dependent mechanisms. 

As regards the other analyses of changes in skeletal gene expression higher Dkk-1 mRNA levels in Nx+PTx 1.2% relative to Nx+PTx 0.6% and sham animals. We still do not know the real role of Dkk-1 on renal osteodystrophy. Another interesting finding was that Gsk3b, which leads to phosphorylation of β-catenin and stimulates β-catenin degradation was lower in CKD animals fed 0.6% phosphate than those fed 1.2% phosphate diet, again suggesting that dietary phosphate is involved in the regulation of Wnt/β-catenin signaling. In addition, phosphate was also shown to increase RANK gene expression, which could equally have contributed to the observed lower bone volume.

Our study has several limitations. First, we did not evaluate inflammatory markers, which could have influenced serum sclerostin levels. Second, serum phosphate levels and phosphate FE were only in fasting state. Third, there was no correlation between the serum levels of sclerostin and SOST gene expression values. A longer observation time might have been necessary to observe increased circulating sclerostin resulting from the increase in SOST mRNA. Another possibility is that there are still technical problems with the determination of serum sclerostin, both in humans and in animals. Fourth, for the analyses of gene expression in bone, a small number of samples was analyzed. Nevertheless, we believe that did not invalidate our results, since the numbers were very uniform with almost no variation within groups and with variation of almost ten times among them [42]. Fifth, for calcitriol measurements we used pools of sera, allowing comparison of only few samples per group which could explain the observed absence of significant differences between groups. Finally, we did not include a control group put on 1.2% phosphate diet, because these animals would almost certainly have developed secondary hyperparathyroidism. 

In conclusion, high as compared to normal dietary phosphate intake reduces bone volume in CKD rats with ABD. We show for first time that in this condition dietary phosphate intake stimulates Wnt pathway suppressors, regulating bone SOST and Dkk-1 mRNA expression independently of PTH. High phosphate intake also increases Gsk3b mRNA and RANK mRNA expression, and enhances osteoblast and osteocyte apoptosis in CKD rats with ABD. The underlying mechanisms require further study. Finally, when trying to extrapolate these findings to the clinical setting, they would tend to underscore the importance of controlling dietary phosphate intake and absorption in patients with CKD and ABD.
